# The variations of wheat–maize production, soil organic carbon, and carbon footprints: insights from a 20–year on–farm observational experiment in the North China Plain

**DOI:** 10.3389/fpls.2025.1547431

**Published:** 2025-04-28

**Authors:** Ning Wang, Zhipin Ai, Qiuying Zhang, Peifang Leng, Yunfeng Qiao, Zhao Li, Chao Tian, Xinjie Shi, Hefa Cheng, Gang Chen, Fadong Li

**Affiliations:** ^1^ ShandongYucheng Agro-Ecosystem National Observation and Research Station, Institute of Geographic Sciences and Natural Resources Research, Chinese Academy of Sciences, Beijing, China; ^2^ Key Laboratory of Ecosystem Network Observation and Modeling, Institute of Geographic Sciences and Natural Resources Research, Chinese Academy of Sciences, Beijing, China; ^3^ College of Resources and Environment, University of Chinese Academy of Sciences, Beijing, China; ^4^ Chinese Research Academy of Environmental Sciences, Beijing, China; ^5^ College of Resources and Environmental, Henan Agricultural University, Zhengzhou, Henan, China; ^6^ The MOE (Ministry of Education) Key Laboratory for Earth Surface Processes, College of Urban and Environmental Sciences, Peking University, Beijing, China; ^7^ Department of Civil & Environmental Engineering, College of Engineering, Florida A&M University-Florida State University, Tallahassee, FL, United States

**Keywords:** climate change, agricultural practice, carbon sequestration, greenhouse gas emissions, long-term field experiment, soil nutrient status

## Abstract

**Introduction:**

Climate change is a substantial threat to the global food supply, especially for the North China Plain (NCP), a critical agricultural region in China that exhibits high sensitivity and vulnerability to climate change. Under climate change, many uncertainties remain regarding crop yields, soil organic carbon (SOC), and greenhouse gas (GHG) emissions.

**Methods:**

A 20−year on−farm observational study (2003−2022) of a winter wheat−summer maize rotation system was conducted to comprehensively quantify the continuous variations in crop productivity, SOC storage, GHG emissions, and carbon footprints (CFs) in the NCP.

**Results:**

A warming trend of 0.08°C per year and an annual increase of 57 hours in sunshine duration were detected over the study period. Both wheat and maize yields showed sustained improvements, with annual rates of 70 kg ha–1 and 184 kg ha–1, respectively. Wheat yields were primarily influenced by cumulative sunshine hours in November and soil total potassium (K) content, whereas maize yields were significantly affected by wheat-season agricultural inputs (water, N, P, K fertilizers) and initial soil properties (pH, N, P, K). Although wheat production generated higher GHG emissions than maize (7,307.5 vs 2,998.7 kg CO2-eq ha−1), the wheat season transitioned into a net carbon sink (CF < 0) due to SOC accumulation (0.58 g kg−1 year−1). Conversely, SOC depletion (-0.72 g kg−1 year−1) during the maize season resulted in a carbon source status (CF > 0). This divergence likely stems from contrasting straw management practices: wheat straw incorporation at 20 cm depth versus maize straw surface mulching.

**Discussion:**

Our findings demonstrate significant improvements in crop yields, SOC sequestration, and net ecosystem economic budget over two decades. However, the decelerating trends in yield gains and SOC accumulation rates warrant strategic attention to sustain long-term agricultural resilience.

## 1 Introduction

The global population is estimated to grow over 9.7 billion by the end of 2050 (Ehrlich et al., 2012; Oluwole et al., 2023), leading to a substantial surge in worldwide food demand (Tilman et al., 2011). Crop production enhancement is the ultimate solution to ensure food security, especially due to the limited arable land (Chen et al., 2014; Ai et al., 2020). However, enhanced food production is generally at an environmental cost (Xia et al., 2023) with increased greenhouse gas (GHG) emissions, either directly or indirectly through increased use of electricity, synthetic fertilizers, pesticides, etc (Benbi, 2018). Currently, agriculture is widely recognized as a major contributor of GHG emissions, accounting for 10%–14% of direct anthropogenic GHG emissions (Montzka et al., 2011). When considering indirect emissions, this figure can escalate to one-quarter to one-third of the total emissions (Scialabba and Müller-Lindenlauf, 2010). Subsequently, a comprehensive understanding of the interplay between crop production and agricultural GHG emissions is paramount for the future agricultural development.

Climate is a dominant factor affecting crop production with intricate effects on key crop processes, and the magnitude and direction of the effects are ambiguous (Walker and Schulze, 2008; Chen et al., 2015; Rezaei et al., 2023). For instance, rising temperatures might lead to the reduction of crop yields through mechanisms such as the induction of irreversible plant physiological damage and soil water stress caused by increased evapotranspiration (Mo et al., 2009; Zhao et al., 2017). On the contrary, rising temperatures might benefit crop production in regions of low temperatures that limit crop growth, as evidenced in areas like North America (Izaurralde et al., 2003) and North China Plain (Rashid et al., 2019). Climate change impacts on crop productivity are thus site-specific, and the conclusions derived from site-specific studies cannot be universally applied to different regions. Besides climate change, local agronomic management practices, including irrigation, fertilization, tillage, etc. play a critical role in influencing crop production (He et al., 2015; Liu et al., 2018a). Challinor et al. (2014) suggested that the impact of climate on crop production could be mitigated through appropriate adaptive management strategies. Some research investigated the impacts of various agronomic practices on crop growth and production in response to climate change using short−term field experimental data of 2 to 5 years (Liu et al., 2021a; Liao et al., 2022). It’s essential to acknowledge that conclusions drawn from these short-term experiments have limitations, particularly since certain management practices exhibit hysteresis effects on agricultural processes. For example, as a widely accepted practice to increase soil organic carbon (SOC), straw returning showed obvious hysteresis effects on SOC (Li et al., 2019). A meta-analysis conducted by Wang et al. (2021) revealed that the impact of straw returning on SOC during 6 to 9 years was almost double that of 1 to 5 years, which weakened after 10 years. Presently, long-term field experiment data are needed to provide credible insights into how crop production responds to climate and continuously adapted agronomic practices on a long-term basis.

Carbon footprint (CF) is commonly used as an indicator to link GHG (mainly CO_2_, N_2_O, and CH_4_) emissions to human activities across various agricultural management scenarios, and to quantify the intensities in terms of carbon dioxide equivalent (CO_2_−eq) (Ozlu et al., 2022). For the quantitative calculation of CF associated with different field operations and cropping systems, the life cycle assessment (LCA) method has been widely used in numerous field experiments (Xue et al., 2016; He et al., 2019; Luo et al., 2021). It’s important to note that, in order to minimize noise effects, these rigorous field trials typically were designed to maintain consistency in common agronomic practices such as irrigation and fertilization across years. However, in practice, these operations were commonly adjusted by farmers based on the actual conditions over the years, leading to differences in CF estimation between field experimental results and farm survey data (Yan et al., 2015). While many researchers primarily focused on CF on area and yield scales, expressed as CF_a_ and CF_y_, respectively (Liu et al., 2018b, 2021a), the income-scaled CF had received relatively less attention (Yang et al., 2014; Chen et al., 2021). Furthermore, to the best of our knowledge, there has been little research that delves into different perspectives of CF based on long-term on–farm experimental data. Therefore, assessing CF on various scales would offer both farmers and policymakers a multi-dimensional view of agricultural production and GHG emissions, which helps interpret scientifically informed and effective strategies for mitigating GHG emissions associated with crop production in response to climate change.

The North China Plain (NCP), one of China’s major granaries, produces 61% wheat (*Triticum aestivum* L.) and 31% maize (*Zea mays* L.) of national food production (Qin et al., 2022). Recent studies have revealed escalating climate-agriculture interactions in the NCP: (1) climate drivers – including a ∼1°C warming during the last century (Zhao et al., 2017), a nearly 15% decrease in seasonal precipitation (Mei et al., 2013), and a decrease rate of 0.2 MJ m^-2^ day^-1^ in solar radiation (Tao et al., 2017); (2) management pressures – exemplified by groundwater depletion rates exceeding 1 m yr^-1^ in intensive irrigation zones (Yang et al., 2015a) and progressive soil organic carbon (SOC) loss (-0.18 Mg C ha^-1^ yr^-1^) under conventional tillage practices (Liu et al., 2014). While existing research has disentangled singular stress impacts – such as the wheat yield reduction induced by drought (Wang et al., 2018) and the maize yield penalty caused by post-anthesis warming (Chen et al., 2018) – there remains a notable gap in integrated assessments of adaptation strategies (dynamics of grain production, SOC, and CFs) for the entire rotation system under compounded climate risks in NCP.

In this study, a comprehensive on–farm experiment was conducted in NCP from 2003 to 2022, and the objectives of this study were: 1) to investigate the long-term responses of crop production to climate conditions, soil characteristics, and human inputs, and to identify the critical influencing factors for wheat and maize yields, and 2) to assess the variations of SOC, total GHG, and CFs throughout long–term maize–wheat rotation.

## 2 Materials and methods

### 2.1 Experimental field description

**Figure 1:**
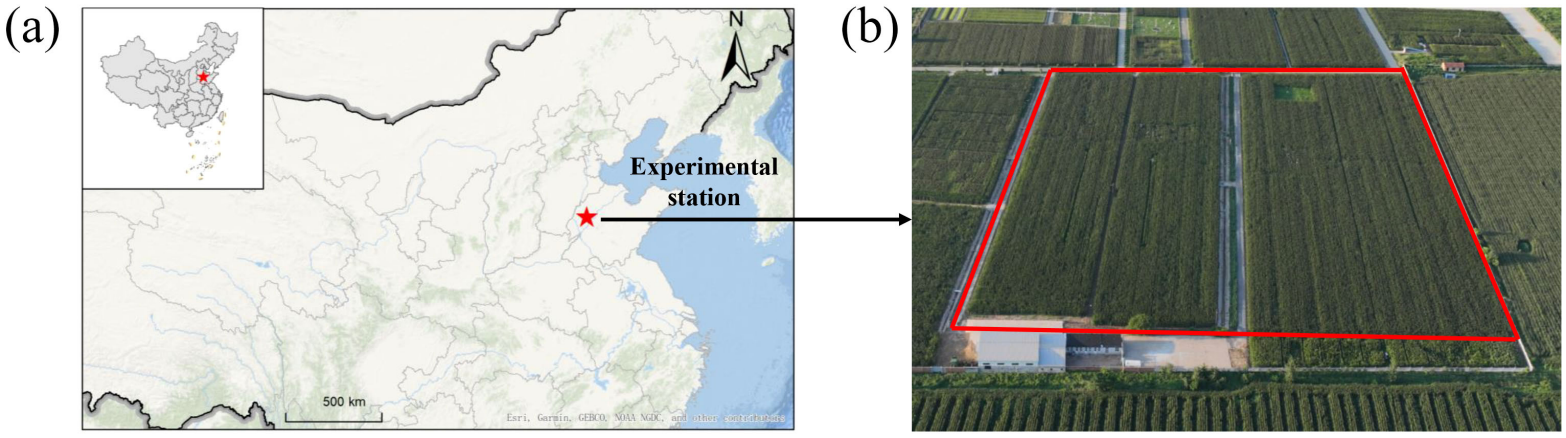
The location of experimental station **(a)** and the experimental field **(b)**.

**Figure 2:**
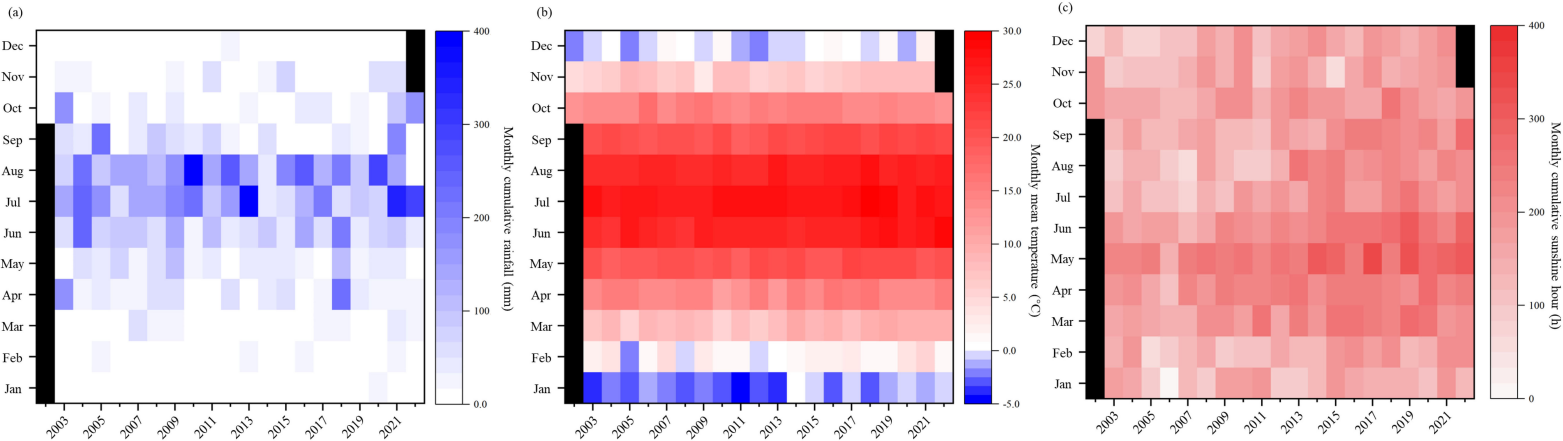
The monthly cumulative rainfall **(a)**, mean temperature **(b)**, and cumulative sunshine hour **(c)** from 2003 to 2022 at Dezhou City, Shandong Province, China.

The long-term experimental site covers 2.2 hectares, and is located at Shandong Yucheng Agro-ecosystem National Observation and Research Station (SYA-NORS), Ministry of Science and Technology in Shandong Province of China (E36°56′N, 116°40′E, 23 m a.s.l) (**Figure 1**). This region has a semi-humid monsoon climate with an average annual temperature of 14.4°C and a mean annual precipitation of 969.6 mm (Li et al., 2023). **Figure 2** illustrates the monthly cumulative rainfall, mean temperature, and cumulative sunshine hours. During the winter wheat growing season (November to May) from 2003 and 2022, the average monthly cumulative rainfall was 19.5 mm, the monthly average mean temperature was 7.0°C, and the average monthly cumulative sunshine hours were 180.3 h. In the case of the maize growing season (June to September), the average monthly cumulative rainfall reached 119.8 mm. The average of monthly mean temperature was 24.9°C. The average monthly cumulative sunshine hours were recorded as 183.5 h. The monthly mean temperature and cumulative sunshine hours during the maize and wheat seasons exhibited a gradual increase trend over the years (**Supplementary Figure S1**).

The field had been under continuous winter wheat–summer maize rotation before the start of the experiment. The soil was classified as salinized tidal soil with main properties (0 – 20 cm depth) as follows: total organic matter, 12.06 ± 1.21 g kg^−1^; total nitrogen, 0.64 ± 0.06 g kg^−1^; total phosphorus, 0.82 ± 0.06 g kg^−1^; available phosphorus, 6.62 ± 2.00 mg kg^−1^; rapidly available potassium, 70.83 ± 4.92 mg kg^−1^; slowly available potassium, 858.33 ± 36.56 mg kg^−1^; and soil bulk density, 1.39 ± 0.08 g cm^−3^.

The experiment was conducted from the beginning of the 2003 winter wheat season to the end of 2022 summer maize season. Winter wheat was generally sown in October and harvested in June of the following year, while summer maize was grown from June to October. The agricultural practices were identical to those of local farmers, including the variety, sowing and harvest date, irrigation frequency and duration, field mechanical operations (i.e., sowing, tillage, and harvesting), herbicide and pesticide applications, and straw returning practices. It should be noted that the topsoil tillage (0–20 cm) was only conducted before winter wheat sowing; therefore, the straw returning method was straw surface mulching for the maize season and straw incorporation for the wheat season. Further detailed descriptions of annual agricultural practices (mainly the time and amount of irrigation, pesticides, and fertilizer, respectively) for winter wheat and summer maize are presented in **Supplementary Tables S1**-**S4**. The amount of straw returned to the field is related to the crop biomass of the previous season, which is shown in **Supplementary Tables S3** and **S4**.

### 2.2 Measurements

#### 2.2.1 Plant parameters

When the plants reached maturity, the measurements of plant height, stem diameter, root dry weight, and aboveground dry weight were conducted in each wheat/maize season, and then, the yields were determined. Twenty maize and wheat plants were randomly selected to measure the plant height (tapeline, m) and stem diameter (vernier caliper, mm) for each plot. For maize, yield data included spike length (ruler, m), cob diameter (vernier caliper, mm), row number (counted), kernels per spike (counted), spike row number (counted), and thousand kernel weight (counted and weighted after air drying). For wheat, yield data included the number of pikes per plant (counted), number of grains per spike (counted), number of fertile spikes per plant (counted), number of spikes per ear (counted), and thousand kernel weight (counted and weighted after air drying). After the above measurements, the plants were cut from the soil line and oven-dried at 85°C until no mass change was observed. The root samples were excavated to a depth of 0.2 m depth in a sampling area of 0.4 m × 0.2 m for winter wheat and 0.6× 0.3 m for summer maize, respectively. The dry root weights were also measured by the oven-dried method. The total dry biomass was the sum of aboveground dry weight and root dry weight.

#### 2.2.2 Soil parameters

Soil organic matter, total nitrogen, total phosphorus, total potassium, available nitrogen, available phosphorus, rapidly available potassium, and pH of topsoil (0–0.2 m) were measured at every harvest season using the same methods as Liu et al. (2023).

Briefly, the SOC was calculated by the following equation: SOC = SOM × 0.58, where 0.58 is the constant coefficient that converts SOM to SOC (Wu et al., 2021).

SOC storage, SCS, was estimated based on SOC, bulk density and soil depth based on Equation 1:


(1) SCS=SOC×BD×H×10

where SCS is the SOC storage (Mg ha^−1^); SOC is the soil organic carbon concentration (g kg^–1^); BD is the soil bulk density (g cm^−3^); H is the thickness of the soil layer (m) (0.2 m in this study); and 10 is the coefficient for converting kg m^−2^ into Mg ha^−1^. Since no significant difference in BD was observed in this study (data not shown), an average value of 1.33 g cm^−3^ was used for BD.

The sequestered rate (C_seqrate_, Mg CO_2_-eq ha^−1^ yr^−1^) was calculated using Equation 2:


(2) $${\text{C}}_{\text{seqrate}}=({\text{SCS}}_{\text{harvest}}-{\text{SCS}}_{\text{preseason}})\times 44/12$$

where SCS_harvest_ and SCS_preseason_ were SOC storage after and before the growing season (Mg CO_2_-eq ha^−1^ yr^−1^), respectively and 44/12 is the coefficient value to convert C to CO_2_.

#### 2.2.3 Grain yield and harvest index

At physiological maturity, the yield was measured using the quadrat method for each plot (1.0 m × 1.0 m for wheat and 3.0 m × 1.0 m for maize). The harvest index was estimated by the ratio of grain yield per plant to aboveground biomass.

#### 2.2.4 Meteorological data

Meteorological parameters including relative humidity, air temperature, wind speed, and photosynthetically active radiation were recorded at 30-minute intervals at an automatic weather station (Model CR23XTD, Campbell Scientific Inc., USA) located 1.8 m above the ground surface near the experimental site (Yue et al., 2023).

### 2.3 Calculation of carbon footprint

The LCA methodology was used to estimate total GHG emissions throughout the whole growth seasons of winter wheat (2006 – 2017 and 2018 – 2020) and summer maize (2005 – 2017 and 2018 – 2020). The system boundaries are shown in **Supplementary Figure S2**. The total GHG emissions (in terms of kg CO_2_-eq) included plant and soil CO_2_ emissions and non-CO_2_ GHG emissions from soil carbon and nitrogen cycling processes (i.e., CH_4_ and N_2_O). N_2_O has a high global warming potential (265 times that of CO_2_ (Masson-Delmotte et al., 2021)), and contributes substantially to CF, while CH_4_ emissions from dryland are negligible due to their minuscule share in total GHG emissions during dryland crop production (He et al., 2019). In this study, N_2_O emissions from soil nitrogen cycling were quantified via emission factors. Equations 3 and 4 were used to calculate *CFa*:


(3) $$CF_{a}=\sum_{\text{n}=1}^{\text{n}}AI_{i}\times EF_{i}+GHG_{N2O}-C_{seqrate}$$

(4) $$GHG_{N_{2}0}=(N_{2}O_{direct}+N_{2}O_{indirect})\times 265$$

where, *CF_a_* (kg CO_2_-eq ha^−1^) is the carbon footprint per area; *AI_i_* is the agricultural inputs shown in **Supplementary Tables S3** (wheat) and S4 (maize); *EF_i_* is the specific GHG emission factor shown in **Supplementary Table S5**; *C_seqrate_* is the soil C sequestered rate (kg CO_2_-eq ha^−1^) from Equation 2; $GHG_{N_{2}0}$ is the total N_2_O emissions (kg CO_2_-eq ha^−1^); *N_2_O_direct_* is the direct N_2_O emissions (kg ha^−1^); *N_2_O_indirect_* is the indirect N_2_O emissions (kg ha^−1^) and 265 is the global warming potential value by the 100−year time horizon (1 for CO_2_) (Masson-Delmotte et al., 2021).

Direct N_2_O emissions were calculated using Equation 5:


(5) $$N_{2}O_{direct}=(F_{Syntheic}+F_{Straw})\times {\text{σ}}_{1}\times 44/28$$

where *F_Syntheic_* is the amount of synthetic fertilizer N input; *F_Straw_* is the amount of N input from crop straw, calculated by multiplying the amount of straw mulching by the N content of straw (N content of wheat & maize straw was determined every year in this study, data not shown); σ_1_ represents the direct emission coefficient of N inputs and 44/28 is the coefficient to convert N_2_ to N_2_O.

The indirect N_2_O emissions were calculated using Equations 6-8:


(6) $$N_{2}O_{indirect}=N_{2}O_{vol}+N_{2}O_{leach}$$

(7) $$N_{2}O_{vol}=(F_{Syntheic}+F_{Straw})\times Frac_{vol}\times {\text{σ}}_{2}\times 44/28$$

(8) $$N_{2}O_{leach}=(F_{Syntheic}+F_{Straw})\times Frac_{leach}\times {\text{σ}}_{3}\times 44/28$$

where *N_2_O_vol_* is the N_2_O emissions from NH_3_ volatilization (kg ha^−1^); *Frac_vol_* is the fraction of N fertilizer volatilized as NH_3_; σ_2_ is the emission factor for the volatilization of N fertilizer; *N_2_O_leach_* is the N_2_O emissions from leaching (kg ha^−1^); *Frac_leach_* is the fraction of N fertilizer leaching; σ_3_ is the emission factor for N leaching and 44/28 is the factor to convert N_2_ to N_2_O.

CF from multiple perspectives was investigated. Specifically, the area-scaled carbon footprint (*CF_a_*) was estimated by Equation 3, the yield-scaled carbon footprint (*CF_y_*, kg CO_2_-eq kg^−1^) was estimated by Equation 9, and the income-scaled carbon footprint (*CF_NEEB_*, kg CO_2_-eq US^−1^) was estimated by Equation 10.


(9) $$CF_{y}=CF_{a}/\text{yield}$$

(10) $$CF_{NEEB}=CF_{a}/NEEB$$

where NEEB is the net ecosystem economic budget (US$ ha^−1^). The NEEB is an indicator to evaluate the economic profits of the cropping system, which can be calculated by Equation 11 (Zhang et al., 2015):


(11) $$\text{NEEB}=Y_{gain}-A_{cost}–C_{cost}$$

where *Y_gain_* is the grain yield gains, calculated by multiplying the crop price for the corresponding year by the crop yield (US$ ha^−1^); *A_cost_* is the sum of the agricultural input costs, consisting of costs associated with mechanical tillage, seeds, fertilizers, electricity, chemical pesticides, and mechanical harvesting (US$ ha^−1^); *C_cost_* is the cost that is calculated by multiplying the C-trade price (US$ 17 ton^−1^ CO_2_-eq) by total GHG emissions (kg CO_2_-eq ha^−1^) from Equation 3, (US$ ha^−1^) (Li et al., 2015).

### 2.4 Data analysis

The one-way analysis of variance (ANOVA) was performed to determine the significance of SOC differences between wheat and maize growing seasons with the least significant difference (LSD) at the level of *p* < 0.05 using SPSS v. 26.0 (IBM, Inc., Armonk, NY, USA). All plots were drawn by Microsoft Excel 2021 (Microsoft Corp., Redmond, WA, USA) and Origin (OriginLab Corp., Northampton, MA, USA). The Mantel analysis and random forest were conducted by R 4.3.1 via packages of “ggcor” and “randomForest”, respectively.

## 3 Results

### 3.1 Wheat and maize yield

**Figure 3:**
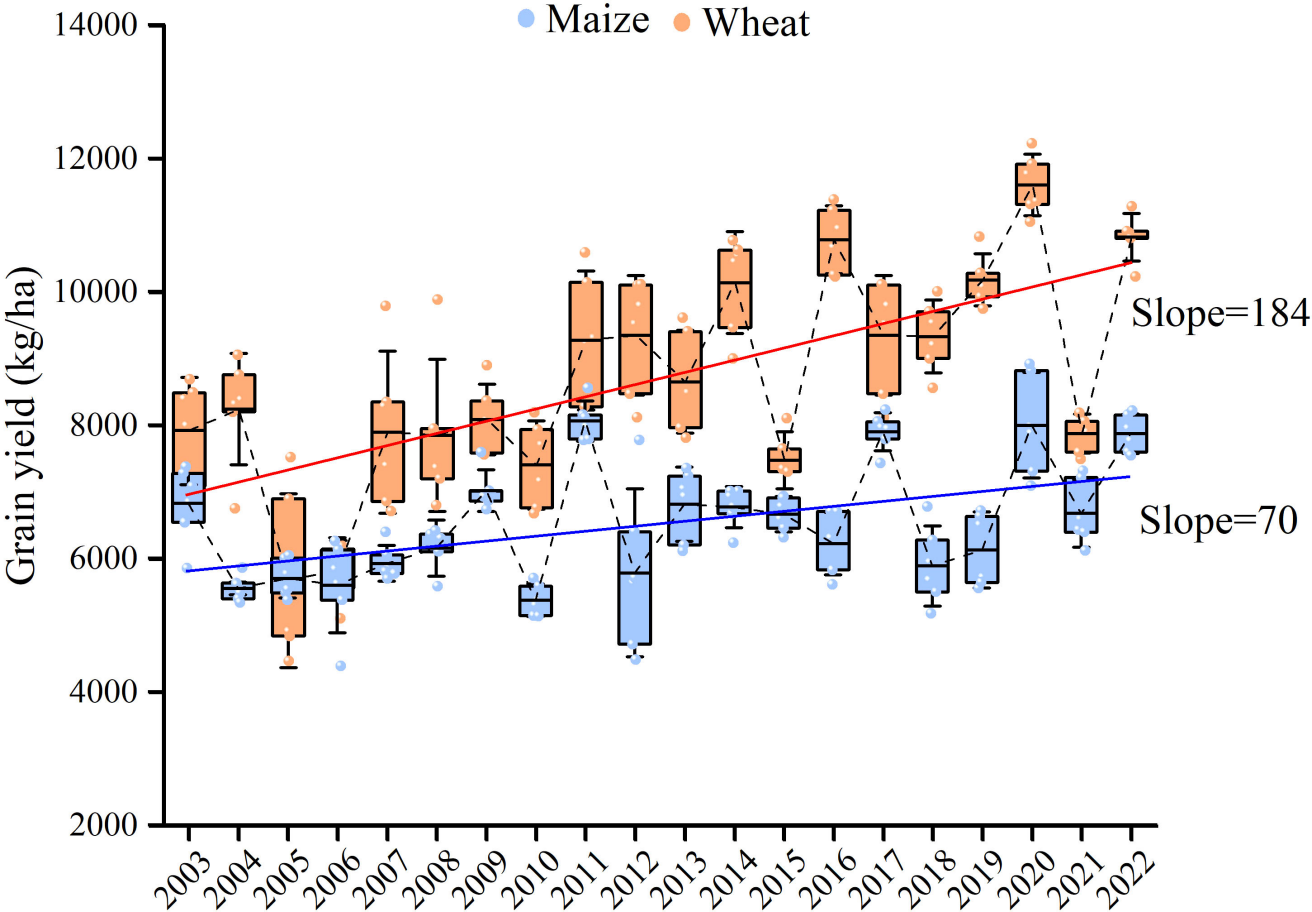
The grain yields of wheat and maize from 2003 to 2022.

The average winter wheat grain yields over the 20-year period were 6,542 kg ha^−1^ (**Figure 3**). Analyzing the 20-year dataset revealed a generally increasing trend in winter wheat production, although the increase rate gradually decreased. The average yields from 2003 to 2007 were 5,918 kg ha^−1^, which increased by 9.4% to 6,474 kg ha^−1^ from 2008 to 2012. However, the yields from 2013 to 2017 were only 6.1% higher than those of 2008 to 2012, and the yields from 2018 to 2022 were just 0.5% greater than those of 2008 to 2012. The average summer maize yields from 2003 to 2022 reached 8,697 kg ha^−1^ (**Figure 3**). Taking a 20-year perspective into account, maize yields showed substantial improvement, although the rate of increase declined over time. Yields for the periods 2002 to 2007, 2008 to 2012, 2013 to 2017, and 2018 to 2022 were 7,123 kg ha^−1^, 8,403 kg ha^−1^, 9,291 kg ha^−1^, and 9,973 kg ha^−1^, respectively, with increasing rates of 18.0%, 10.6%, and 7.3% in succession.

### 3.2 Mantel analysis

**Figure 4:**
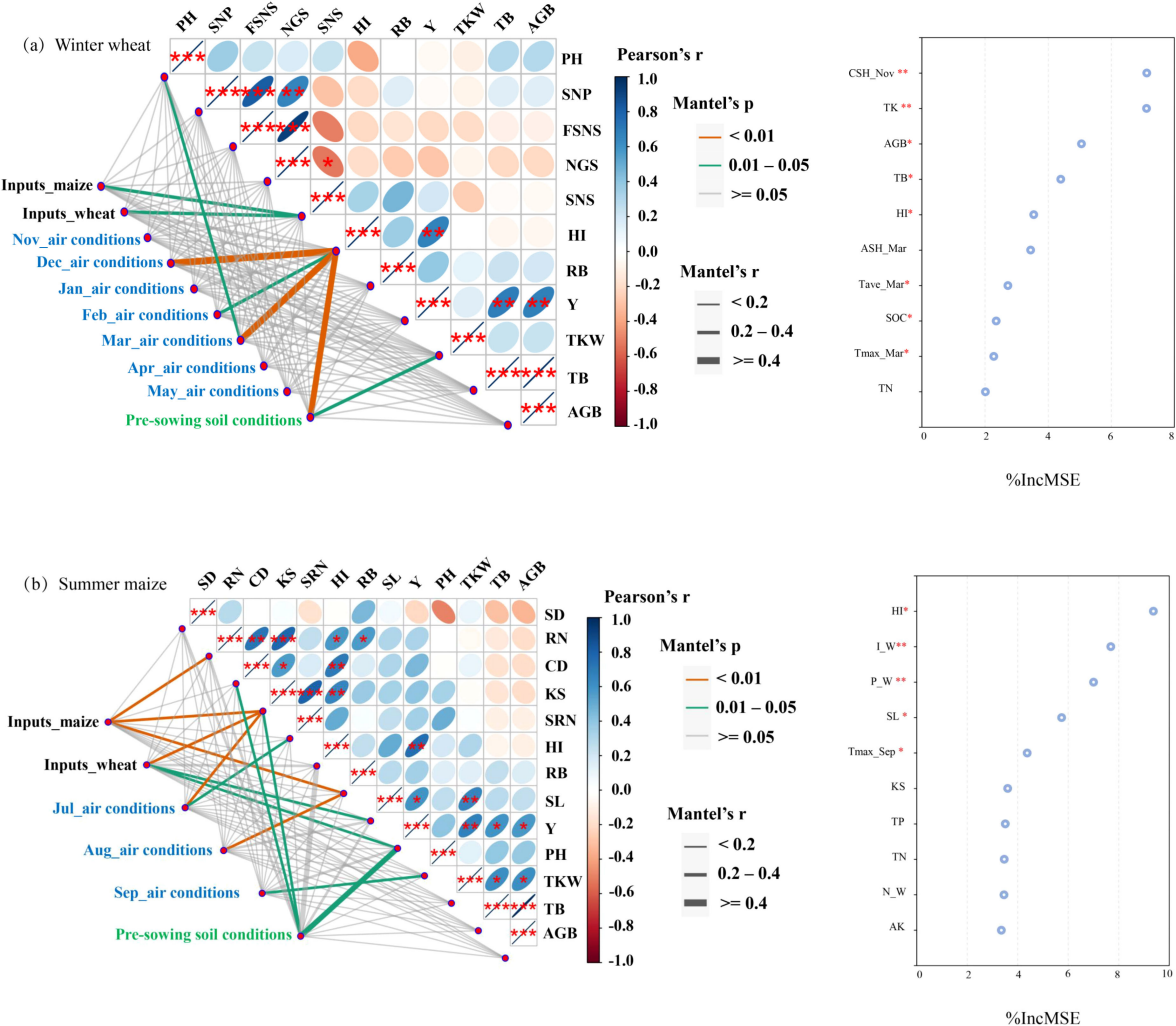
The mantal test and random forest analysis for wheat **(a)** and maize **(b)**. The Mantel test primarily explored: 1) the correlation between plant growth traits and yield components; and 2) the main factors affecting growth traits and yield composition. The random forest analysis was used to identify the key driving factors affecting grain yield. The inputs include the application of irrigation (I) and N, P, and K fertilizers for both wheat (W) and maize (M) seasons (November to May for wheat and July to September for maize); The air conditions include average air temperature (T_ave_), maximum air temperature (T_max_), minimum air temperature (T_min_), cumulative rainfall (AR), and cumulative sunshine hours (CSH); Soil conditions include soil organic carbon, pH, total nitrogen (TN), total phosphorus (TP), total potassium (TK), available nitrogen (AN), available phosphorus (AP), and rapidly available potassium (RAK); PH means plant height; SNP means spikelets number per spike (wheat); FSNS means fertile spikelets number spike (wheat); NGS means number of grains per spike (wheat); SNS means spikelets per spike (wheat); SD means stem diameter (maize); RN means row number (maize); CD means cob diameter (maize); KS means kernels per spike (maize); SRN means spike row number (maize); SL means spike length (maize); HI means harvest index; RB means root biomass; Y means yield; TKW means thousand kernel weight; TB means total biomass; AGB means aboveground biomass. The significance was marked with asterisks: * P<0.05, ** P<0.01, and *** P<0.001.

In the case of winter wheat, there was a significant positive relationship between wheat yields and harvest index, aboveground biomass, and total biomass (**Figure 4a**). Additionally, significant positive correlations were observed among spikelets per spike, fertile spikelets per spike, and the number of grains per spike. Conversely, a significant negative relationship was found between spikelet number per spike and the number of grains per spike. For maize plant (**Figure 4b**), the yields exhibited a positive correlation with harvest index and spike length. Row number was positively related to kernels per spike, cob diameter, harvest index, and root biomass. Both cob diameter and kernels per spike showed significant correlations with harvest index. Additionally, significant correlations were detected between thousand kernel weight and spike length, total biomass, and aboveground biomass.

For the wheat plant, the harvest index was significantly affected by air conditions in December, February, and March, as well as pre-sowing soil conditions. The number of spikelets per spike was influenced by inputs from both wheat and maize seasons, and pre−sowing soil conditions played a critical role in determining thousand kernel weight. Maize yields were significantly affected by pre-sowing soil conditions and seasonal wheat inputs. Seasonal maize inputs had significant effects on row number, kernels per spike, and root biomass, whereas seasonal wheat inputs significantly influenced kernels per spike and spike length, in addition to yields. July air conditions significantly affected the kernels per spike and spike row number. Root biomass and plant height were influenced by air conditions in August and September, respectively. Pre−sowing soil conditions had significant effects on cob diameter and kernels per spike, in addition to yields.

The top ten key factors affecting wheat and maize yields (**Figure 4**) were selected by using the random forest method. The accumulative sunshine hours in November ranked as the most critical factor, followed by soil total K, aboveground biomass, total biomass, harvest index, accumulative sunshine hours in March, soil organic carbon, average air temperature in March, and total nitrogen. Among these ten factors, four were related to the air condition matrix, three were associated with the pre-sowing soil condition matrix, and the remaining three factors were harvest index, aboveground biomass, and total biomass. Harvest index was identified as the most significant factor influencing maize yields, followed by the seasonal irrigation and P fertilizer applications for wheat. These were followed by spike length, maximum air temperature in September, kernels per spike, total P and N, seasonal N application for wheat, and available K. Among these factors, three were related to wheat seasonal inputs, three were associated with yield components, three were part of soil conditions, and one was a parameter in the air condition matrix.

### 3.3 Carbon footprints

**Figure 5:**
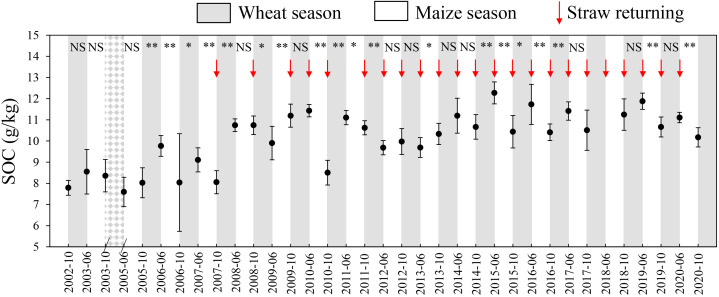
The changes of soil organic carbon (SOC) from October 2002 to October 2020 (without June and October in 2004 and June in 2018). The significance between two adjacent SOC contents was marked with asterisks: NS P>0.05, * P<0.05, and ** P<0.01.

The SOC from October 2002 to October 2020 is presented in **Figure 5**. The straw returning was introduced in October 2007 for the wheat season and in July 2010 for the maize season (with the exception of July 2011). Before October 2007, the average SOC was 8.4 g kg^−1^, which increased by 26%, reaching 10.6 g kg^−1^ after the application of straw returning. Over the 20-year period, an increasing trend in SOC was observed during the winter wheat season, rising from 16.8 g kg^−1^ to 18.3 g kg^−1^. In contrast, the SOC decreased by 6.3% from 18.0 g kg^−1^ to 16.9 g kg^−1^ during the maize growing season.

Fourteen and fifteen growing cycles were used to calculate grain yield gains, agricultural inputs, total GHG emissions, and NEEB for the wheat and maize seasons, respectively (**Table 1**). The average income for wheat was $2,183 ha^−1^, slightly lower than that of maize, which was $2,368 ha^−1^. Wheat seasonal agricultural inputs were $814 ha^−1^, exceeding that of the maize season by $238 ha^−1^. Wheat seasonal total GHG emissions ranged from 5,145 kg CO_2_-eq ha^−1^ in 2009 to 8,047 kg CO_2_-eq ha^−1^ in 2007, with an average of 7,307 kg CO_2_-eq ha^−1^. Maize seasonal total GHG emissions were significantly lower than those of wheat, ranging from 1,721 kg CO_2_-eq ha^−1^ in 2011 to 5,707 kg CO_2_-eq ha^−1^ in 2014. The NEEB for the wheat season averaged at $1,244 ha^−1^, which was $1,741 ha^−1^ for the maize season. The total GHG emissions showed no significant fluctuation over time in either wheat or maize season. However, grain yield gains, agricultural inputs cost, and NEEB all exhibited an increase trend over years for both crops. Grain yield gains increased by $100 ha^−1^ annually for wheat and $148 ha^−1^ annually for maize. In terms of NEEB, there was an annual increase of $83 ha^−1^ for the wheat season and $120 ha^−1^ for the maize season.

**Table 1:** The grain income (US$ ha^–1^), cost of agricultural input (US$ ha^–1^), GHG emissions (kg CO_2_-eq ha^−1^), and net ecosystem economic budget (NEEB) (US$ ha^–1^) for wheat (2006 to 2020 except for 2018) and maize (2005 to 2020 except for 2018).

Year	Wheat				Maize			
	Grain income	Agricultural inputs	GHG emissions	NEEB	Grain income	Agricultural inputs	GHG emissions	NEEB
2005					743	229	2986	463
2006	1125	556	7978	433	1054	354	3217	646
2007	1274	627	8047	510	1789	423	5361	1275
2008	1454	662	7034	672	1562	322	3044	1188
2009	2155	807	5144	1261	2063	360	1610	1675
2010	1650	680	7318	845	2060	530	1890	1498
2011	2570	865	7120	1584	2960	481	1721	2450
2012	2234	1088	7701	1015	3203	1101	4236	2030
2013	2770	966	7407	1677	2881	859	3143	1969
2014	2732	892	7323	1716	3503	798	5707	2607
2015	2437	944	8027	1357	1899	590	2554	1266
2016	2454	832	7439	1495	2438	738	2504	1657
2017	2924	878	7473	1919	2413	520	2120	1857
2019	2095	765	6971	1211	2747	681	2124	2031
2020	2688	838	7322	1725	4210	658	2762	3505
Average	2183	814	7307	1244	2368	576	2999	1741

**Figure 6:**
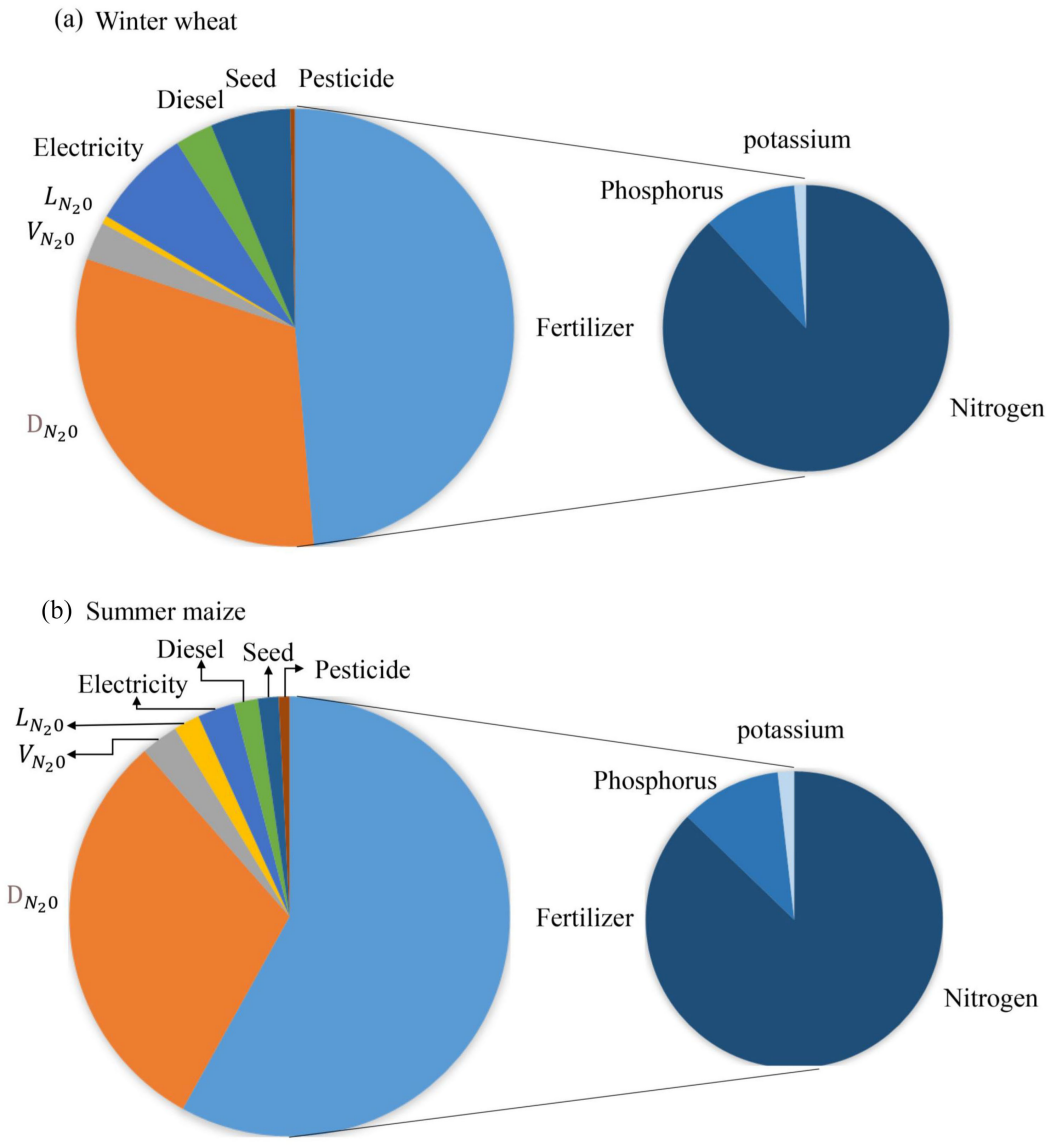
Component of total greenhouse gases emissions for wheat **(a)** and maize **(b)**. $D_{N_{2}0}$ means the direct emissions from; $L_{N_{2}0}$ means the N_2_O emissions from leaching; $V_{N_{2}0}$ means the N_2_O emissions from NH_3_ volatilization.

The components of total greenhouse gas emissions are presented in **Figure 6**. During the wheat season, the largest proportion of total GHG emissions (49%) was attributed to fertilizers, with direct N_2_O emissions accounting for 31%. Similarly, for the maize season, 89% of the total GHG emissions were attributed to fertilizers and direct N_2_O emissions. Emissions from fertilizers were predominantly from nitrogen, accounting for 88% to 87% for wheat and maize growing seasons. Other components, such as electricity (7%) and seeds (6%), also contributed significantly, exceeding 5% of the total GHG emissions for wheat. In addition to fertilizers and direct N_2_O emissions, indirect N_2_O emissions from volatilization and leaching, electricity, diesel, seeds, and pesticides were all in the range of 1% to 3%, summing up to 12% during the entire maize season.

**Figure 7:**
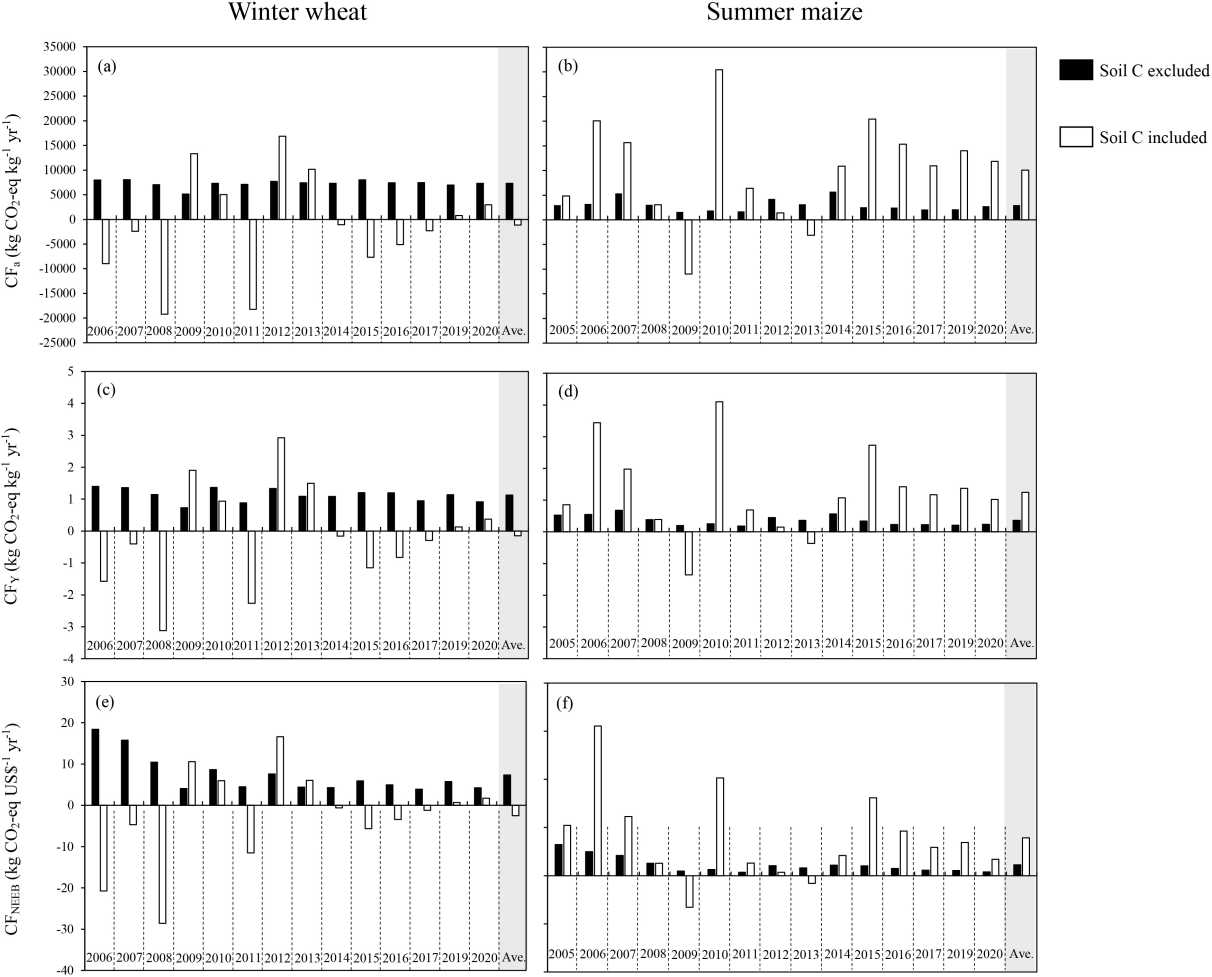
The area-scaled carbon footprint (CFa) for winter wheat **(a)** and summer maize **(b)**, the yield-scaled carbon footprint (CFY) for winter wheat **(c)** and summer maize **(d)**, and NEEB-scaled carbon footprint (CFNEEB) for wheat **(e)** and maize **(f)**.

The values for CF_a_, CF_Y_, and CF_NEEB_, both with and without considering SOC changes, are all presented in **Figure 7**. CF_a_ is equivalent to total GHG emissions when SOC changes are excluded. The average CF_a_ with SOC changes was -1,127 kg CO_2_−eq kg^−1^ yr^−1^ for wheat seasons, and it reached 10,079 kg CO_2_−eq kg^−1^ yr^−1^ for maize seasons. When the SOC changes were not considered, the average CF_Y_ was 1.1 kg CO_2_−eq kg^−1^ yr^−1^ and 0.36 kg CO_2_−eq kg^−1^ yr^−1^ for wheat and maize, respectively. The average CF_Y_ was −0.14 kg CO_2_−eq kg^−1^ yr^−1^ for wheat and 1.24 kg CO_2_−eq kg^−1^ yr^−1^ for maize. Excluding SOC changes, the average of CF_NEEB_ for wheat was 7.4 kg CO_2_−eq US$^−1^ yr^−1^, which was higher than that of 2.2 kg CO_2_−eq US$^−1^ yr^−1^ for maize. With SOC changes included, the average CF_NEEB_ was −2.5 kg CO_2_−eq US$^−1^ yr^−1^ for wheat and 7.8 kg CO_2_−eq US$^−1^ yr^−1^ for maize.

## 4 Discussion

### 4.1 The crop yields and its driving factors

The grain yields of wheat and maize in NCP have been continuously increasing since the 1960s (Wang et al., 2012). Our study demonstrated an improvement in wheat and maize yields from 2003 to 2022, at an annual increase rate of 70 kg ha^–1^ and 184 kg ha^–1^ respectively (**Figure 3**). Similarly, Yu et al. (2022) reported an increase rate of 130 kg ha^–1^ year^–1^ for wheat and 80 kg ha^–1^ year^–1^ for maize from 2003 to 2015. It is worth noting that these inspiring grain yield records were achieved under a linear upward trend with mean temperature (0.08°C year^−1^) and cumulative sunshine hours (57 h year^−1^) (**Supplementary Figure S1**). In general, higher temperatures and increased sunshine hours could advance flowering and maturity of crops, potentially shortening the growing period (Mo et al., 2009) and leading to reduced solar radiation and yield losses (Chen et al., 2013; Huang et al., 2018). However, a steady growing period length was observed for both maize (105 ± 12 days) and wheat (235 ± 15 days), which could be attributed to the continuous adoption of new crop varieties (**Supplementary Tables S6**, **S7**). Liu et al. (2010) proved that the varietal changes, a type of autonomous adaptation response to climate change, significantly helped stabilize the duration of key growth periods for both wheat and maize, mitigating the impact of climate change in NCP. Xiao and Tao (2016) also suggested that the shift from traditional to modern cultivars could offset the negative effects of climate change on maize yields in NCP. These results underscored the importance of considering the combined impacts of climate change and agronomic practices on crop production (He et al., 2020), implying that the potentially adverse effects of climate change on crop productivity may have been overestimated (Asseng et al., 2019; Liu et al., 2021b; Xiao et al., 2020). However, a decrease in the increase rate of wheat and maize yields was highly noticed (**Figure 3**). Ju et al. (2009) attributed this “stable yield period” to the relatively stabilized varieties and management options, and the gradually “saturated” water and fertilizer inputs. This could be partly verified in this study, showing a slowing increase trend of soil N, P, and K (**Supplementary Figure S3**).

It was identified that the cumulative sunshine hours in November played a crucial role in affecting wheat yields, with pre-sowing soil total K concentration being the second most important factor (**Figure 4**). Similarly, Zhuang et al. (2018) also established a significant relationship between wheat yields and sunshine duration during early crop growth stages (i.e., seeding, emergence, and tillering) across NCP. Besides, the substantial impact of soil K on wheat yields was well-documented, primarily attributed to its role in improving photosynthesis, facilitating photosynthate translocation, and reducing grain sterility (Ma et al., 2019; Wang et al., 2020). It is important to note that the preceding crop type could substantially affect the subsequent crop performance (Kuerban et al., 2023; Qin et al., 2023), especially in NCP, where most N fertilizers, irrigation, and all P and K fertilizers were applied before the wheat season for the local wheat–maize rotation system. Therefore, pre-sowing soil conditions and inputs during the wheat season were found to have the most significant effects on maize yields in this study. Wei et al. (2023) reported that the water management for the preceding wheat did not affect the subsequent maize yields but increased the interannual yield variability. Our results further implied the stronger response of maize yields to integrated inputs of irrigation and fertilizers (N, P, and K) during wheat season. Furthermore, our results emphasized the importance of P fertilization for maize production. Other studies, such as Rafiullah et al. (2020) and Chen et al. (2022), also suggested that P availability was a vital but limiting macronutrient, and played a critical role in regulating photosynthesis, carbohydrate metabolism, and root physiological adaptations in crops.

### 4.2 Total GHG emissions, SOC, NEEB, and CFs

The GHG emissions in this study were 7,307 kg CO_2_-eq ha^−1^ for wheat and 2,999 kg CO_2_-eq ha^−1^ for maize (**Table 1**). The largest contributor was fertilizer applications, accounting for 49% to 58% of the emissions (**Figure 6**). These findings aligned with the results of the study by Yang et al. (2014), which demonstrated that fertilizer applications were the primary contributor to GHG emissions (45%) in NCP. When considering both direct and indirect emissions, fertilizers were responsible for a substantial portion of total GHG emissions, contributing to 84% and 94% of total emissions in wheat and maize production, respectively (**Figure 6**). These percentages were consistent with the ranges reported in other published articles (Yang et al., 2014; Cheng et al., 2015), which emphasized the significant role of fertilizers in GHG emissions. Increasing SOC was regarded as a sustainable practice to reduce GHG emissions from agricultural systems, with the potential to sequester 2 to 3 billion metric tons of carbon per year globally (Lenka and Lal, 2013). Interestingly, for SOC, this study found an increase of 0.58 g kg^–1^ year^–1^ for wheat season and a decrease of 0.72 g kg^–1^ year^–1^ for maize after 2011, after the straw returning technique was applied (**Figure 5**). This difference could be attributed to the specific agricultural management in NCP: mechanical tillage was conducted before wheat sowing but not for the maize season. The combination of tillage and straw returning practices (straw incorporation at 20 cm depth) provided a significant amount of organic matter. The increased organic matter enhanced soil microbial activity (Mohamed et al., 2021), promoted the formation of soil humus components (Liu et al., 2020), and increased the amount of root exudates (Yang et al., 2021), all contributing to the preservation and retention of organic carbon in the soil. On the other hand, when only straw returning (surface mulching) was implemented, there was a risk of increasing soil GHG emissions, arising from high soil mineralization and respiration under favorable hydrothermal conditions (Lenka and Lal, 2013; Wang et al., 2023). These findings underlined the complex interplay of agricultural practices and their impacts on GHG emissions in the context of sustainable agriculture. Besides, our study demonstrated that both the increase and decrease of SOC during wheat and maize seasons, presented an “increase-decrease” trend from 2012 to 2020 (**Figure 7**). The gradually decelerated increase-rate of SOC during the wheat season might be caused by the fact that the SOC contents would reach an equilibrium after continuous increase (Yang et al., 2015b), and the time to achieve equilibrium differed depending on the soil conditions of the start of the experiment and the amounts of fertilizers used in the studies. For example, a similar trend, but different decrease scales, was observed by Yang et al. (2015b) based on a 10-year study and by Tong et al. (2009) based on a 17-year study. The gradually decomposing straw might compensate the C loss during the maize season, slowing down the decrease of SOC (Battaglia et al., 2021).

In this study, the NEEB increased for both wheat ($83 ha^–1^ year^–1^) and maize ($120 ha^–1^ year^–1^) over the years, mainly due to the increased grain income ($100 ha^–1^ year^–1^ for wheat and $148 ha^–1^ year^–1^ for maize) (**Table 1**). However, comparing NEEB between wheat and maize is challenging because most agricultural inputs, primarily fertilizers, were applied during the wheat season. When the changes of SOC were included, CFs of maize became positive, indicating that maize production was a carbon source, whereas for wheat, it became negative, suggesting that wheat acted as a carbon sink. These results were consistent with the findings of Gan et al. (2012), who also observed a shift in CF for wheat from positive to negative when SOC changes were included. However, some studies reported that a wheat-maize rotation system was a net carbon source, even considering SOC changes (Yang et al., 2014; Liu et al., 2021a; Yang et al., 2023). The role of the soil in the carbon cycle of agroecological systems remains a subject of debate, but the fundamental importance of SOC and its changes in influencing carbon cycles is widely acknowledged (Ostle et al., 2009; Ogle et al., 2023).

### 4.3 Limitations and potential for future study

This long-term field observational experiment primarily investigated the effects of climate change and field management practices on crop production, SOC, and CFs. However, there are some limitations that should be addressed in future studies: (1) This study focused on only one planting pattern at the field scale. Some research has shown that the effects of climate change on different planting patterns in the North China Plain may exhibit multiple degrees and directions (Yan and Du, 2023). Therefore, future long-term studies should include multiple planting patterns and agricultural management strategies. (2) Only surface SOC (0–20 cm in this study) was measured in this study. Although surface SOC is a key and active component of the terrestrial carbon pool, it has been shown that deep SOC (60–100 cm) is also influenced by climatic conditions (Wen et al., 2023). Hence, future research should examine changes in deep SOC, and reassess soil carbon sequestration and the carbon footprint.

## 5 Conclusions

This study conducted a 20–year on–farm experiment in NCP, aiming to analyze wheat and maize yield responses to environmental factors and agricultural practices, and assess the variations of SOC, total GHG emissions, and CFs. The results showed that although the temperature kept rising across 20 years, both wheat and maize yields exhibited a positive increase trend. This suggested that the renewal of cultivars might offset the negative effects of climate change on grain yields in this region. During the wheat season, there was an increase in SOC at a rate of 0.58 g kg^–1^ per year. In contrast, the maize season resulted in a loss of SOC at a rate of 0.72 g kg^–1^ per year. As a result, despite the fact that GHG emissions generated by wheat production were more than double those of maize, maize production acted as a carbon source, whereas wheat production acted as a carbon sink. The different straw returning techniques, i.e., straw incorporation (20 cm depth) for wheat and straw surface mulching for maize, could partly explain these interesting results. However, due to the gradually “saturated” agricultural inputs and soil nutrition conditions, more attention should be paid to the gradually decelerated increase rates of yield (for both wheat and maize) and SOC contents in the future.

## Data Availability

The original contributions presented in the study are included in the article/**Supplementary Material**. Further inquiries can be directed to the corresponding authors.
