# Supplementary material for: The variations of wheat–maize production, soil organic carbon, and carbon footprints: insights from a 20–year on–farm observational experiment in the North China Plain
Source: Front Plant Sci. 2025 Apr 28;16:1547431. doi: 10.3389/fpls.2025.1547431 (PMC12066458; doi:10.3389/fpls.2025.1547431)

Table S1 The time of major field operations during wheat seasons from 2003 to 2022.

| Year | Fertilizer | | Irrigation | Pesticides |
| --- | --- | --- | --- | --- |
|  | Base fertilizer | Topdressing |  |  |
| 2003 | 10/10/2002 | 03/29/2003 | - | 05/10/2003 & 03/22/2003 |
| 2004 | 10/13/2003 | 03/13/2004 | 03/13/2004 & 04/17/2004 | 03-24-2004 & 05-04-2004 & 05-09-2004 |
| 2005 | 10/08/2004 | 03/30/2005 | 03/30/2005 & 05/02/2005 | 04/10/2005 & 05/18/2005 |
| 2006 | 10/25/2005 | 03/25/2006 | 3/29/2006 & 4/24/2006 | 4/2/2006 & 5/18/2006 |
| 2007 | 10/06/2006 | 03/27/2007 | 11/23/2006 & 03/28/2007 & 05/03/2007 | 03/15/2007 & 05/12/2007 |
| 2008 | 10/21/2007 & 10/22/2007 | 03/13/2008 & 04/18/2008 | 03/13/2008 & 04/18/2008 | 03/15/2007 & 05/12/2007 |
| 2009 | 10/09/2008 & 10/11/2008 | 03/25/2009 | 12/06/2008 & 03/25/2009 | 03/28/2008 & 05/14/2008 |
| 2010 | 10/13/2009 | 04/01/2010 | 04/01/2010 & 05/09/2010 | 03/17/2009 & 04/24/2009 & 05/17/2009 |
| 2011 | 10/10/2010 & 10/14/2010 | 03/24/2011 | 12/05/2010 & 03/25/2011 & 05/04/2011 | 04/08/2011 & 05/13/2011 |
| 2012 | 10/10/2011 & 10/16/2011 | 04/16/2012 | 12/05/2011 & 04/11/2012 | 03/28/2012 & 05/07/2012 & 05/16/2012 |
| 2013 | 10/10/2012 & 10/11/2012 | 04/01/2013 | 04/02/2013 | 03/14/2013 & 05/19/2013 |
| 2014 | 10/17/2013 | 03/18/2014 | 03/19/2014 & 04/17/2014 | 03/28/2014 & 05/07/2014 |
| 2015 | 10/17/2014 & 10/22/2014 | 03/08/2015 | 03/09/2015 | 03/25/2015 & 05/09/2015 |
| 2016 | 10/02/2015 & 10/19/2016 | 03/25/2016 | 03/25/2016 | 03/25/2016 & 05/10/2016 |
| 2017 | 10/09/2016 & 10/11/2016 | 03/22/2017 | 03/24/2017 | 11/17/2016 & 05/05/2017 |
| 2018 | 09/302017 & 10/18/2017 | 03/20/2018 | 03/20/2018 | 02/27/2018 & 05/04/2018 |
| 2019 | 09/29/2018 & 10/09/2018 | 03/09/2019 | 03/09/2019 | 02/25/2019 & 05/18/2019 |
| 2020 | 09/28/2019 & 10/17/2019 | 03/20/2020 | 03/20/2020 | 03/04/2020 & 04/29/2020 |
| 2021 | 09/30/2020 & 10/11/2020 | 02/28/2021 | 03/10/2021 | 03/29/2021 & 05/10/2021 |
| 2022 | 10/02/2021 & 10/11/2021 | 03/29/2022 | 03/29/2022 | 03/09/2022 & 05/15/2022 |

Table S2 The time of major field operations during maize seasons from 2003 to 2022.

| Year | Fertilizer | | Irrigation | Pesticides |
| --- | --- | --- | --- | --- |
|  | Base fertilizer | Topdressing |  |  |
| 2003 | - | 08/05/2003 | - | 06/25/2003 |
| 2004 | - | 07/01/2004 & 07/26/2004 | - | 06-21-2004 & 08-25-2004 |
| 2005 | - | 07/25/2005 | 06/24/2005 | 06/29/2005 |
| 2006 | - | 07/19/2006 | 7/25/2006 | 6/21/2006 |
| 2007 | - | 07/14/2007 & 07/22/2007 | 06/17/2007 | 06/19/2007 & 08/20/2007 |
| 2008 | - | 07/29/2008 | - | 06/22/2008 & 07/12/2008 |
| 2009 | - | 07/16/2009 & 07/26/2009 | - | 06/17/2009 & 07/22/2009 |
| 2010 | 06/17/2010 | 07/22/2010 | - | 06/21/2010 & 07/23/2010 & 08/10/2010 |
| 2011 | - | 06/22/2011 & 08/01/2011 | - | 06/27/2011 |
| 2012 | 06/07/2012 | 06/17/2012 & 08/02/2012 | - | 06/18/2012 & 07/05/2012 |
| 2013 | 06/14/2013 | 06/15/2013 & 07/14/2013 | - | 06/16/2013 & 07/21/2013 |
| 2014 | 06/10/2014 | 06/17/2014 & 07/30/2014 | - | 06/19/2014 |
| 2015 | 06/11/2015 | 06/18/2015 | 06/25/2015 | 07/01/2015 |
| 2016 | 06/07/2016 | 06/19/2016 | - | 07/03/2016 & 07/14/2016 |
| 2017 | 06/07/2017 | 06/17/2017 | - | 06/30/2017 |
| 2018 | 06/07/2018 | 06/15/2018 | - | 07/01/2018 |
| 2019 | 06/11/2019 | 06/15/2019 | - | 07/05/2019 |
| 2020 | 06/06/2020 | 06/16/2020 | 06/17/2020 | 07/05/2020 |
| 2021 | 06/07/2021 | 06/20/2021 | - | 07/06/2021 |
| 2022 | 06/12/2022 | 06/19/2022 | - | 07/06/2022 |

Table S3 The amounts of agricultural inputs during wheat growing seasons from 2003 to 2022.

| Year | Chemical fertilizer  (kg ha^-1^) | | |  | Maize straw*  (kg ha^-1^) | | | | Irrigation  (mm) | Diesel**  (kg ha^-1^) | Seeding rate  (kg ha^-1^) | Pesticides  (kg ha^-1^) |
| --- | --- | --- | --- | --- | --- | --- | --- | --- | --- | --- | --- | --- |
|  | N | P_2_O_5_ | K_2_O |  | Amount | N | P | K |  |  |  |  |
| 2003 | 402.5 | 257.7 | 90.4 |  | 0 | 0 | 0 | 0 | 300.0 | 45.5 | 187.5 | 5.5 |
| 2004 | 349.5 | 257.7 | 90.4 |  | 0 | 0 | 0 | 0 | 300.0 | 45.5 | 225.0 | 2.3 |
| 2005 | 271.5 | 178.6 | 85.0 |  | 0 | 0 | 0 | 0 | 300.0 | 45.5 | 225.0 | 0.0 |
| 2006 | 280.5 | 279.4 | 186.2 |  | 0 | 0 | 0 | 0 | 300.0 | 45.5 | 525.0 | 0.5 |
| 2007 | 327.0 | 137.4 | 72.3 |  | 0 | 0 | 0 | 0 | 385.0 | 45.5 | 225.0 | 3.8 |
| 2008 | 240.0 | 237.0 | 38.0 |  | 8500 | 61.2 | 5.6 | 305.9 | 232.5 | 45.5 | 157.5 | 3.9 |
| 2009 | 105.0 | 155.1 | 37.5 |  | 5680 | 77.0 | 6.1 | 158.0 | 240.0 | 45.5 | 210.0 | 5.0 |
| 2010 | 276.0 | 0.0 | 0.0 |  | 8100 | 109.8 | 8.8 | 225.3 | 225.0 | 45.5 | 262.5 | 1.5 |
| 2011 | 244.5 | 88.6 | 36.0 |  | 7146 | 62.3 | 16.8 | 106.4 | 345.0 | 45.5 | 300.0 | 2.3 |
| 2012 | 312.0 | 57.7 | 47.3 |  | 6068 | 52.9 | 14.3 | 90.4 | 240.0 | 45.5 | 262.5 | 2.3 |
| 2013 | 292.5 | 120.1 | 18.8 |  | 6269 | 65.7 | 5.3 | 63.9 | 97.5 | 45.5 | 300.0 | 1.7 |
| 2014 | 286.5 | 31.0 | 36.0 |  | 5649 | 59.2 | 2.0 | 47.8 | 240.0 | 45.5 | 277.5 | 0.8 |
| 2015 | 338.4 | 59.1 | 60.0 |  | 6910 | 72.4 | 5.8 | 70.5 | 135.0 | 45.5 | 300.0 | 0.4 |
| 2016 | 288.0 | 100.5 | 90.0 |  | 7113 | 68.6 | 5.8 | 125.1 | 135.0 | 45.5 | 300.0 | 2.4 |
| 2017 | 289.5 | 53.2 | 45.0 |  | 10172 | 98.1 | 8.3 | 178.9 | 120.0 | 45.5 | 300.0 | 4.9 |
| 2018 | 160.5 | 106.4 | 40.5 |  | 6269 | 69.7 | 5.3 | 77.0 | 120.0 | 45.5 | 262.5 | 4.2 |
| 2019 | 249.0 | 88.6 | 0.0 |  | 8783 | 97.7 | 7.5 | 108.0 | 150.0 | 45.5 | 262.5 | 3.9 |
| 2020 | 268.5 | 118.2 | 36.0 |  | 8488 | 94.4 | 7.2 | 104.3 | 150.0 | 45.5 | 262.5 | 4.8 |
| 2021 | 280.5 | 130.0 | 30.0 |  | 11410 | 124.8 | 11.2 | 164.5 | 150.0 | 45.5 | 262.5 | 4.2 |
| 2022 | 232.5 | 110.8 | 75.0 |  | 7380 | 79.7 | 7.2 | 105.0 | 150.0 | 45.5 | 300.0 | 6.8 |

Notes: * The straw used to determine the element contents came from the preceding maize season; ** Mechanical tillage and harvesting accounted for 30 kg ha^-1^ and 15.5 kg ha^-1^, respectively.

Table S4 The amounts of agricultural inputs during maize growing seasons from 2003 to 2022.

| Year | Chemical fertilizer  (kg ha^-1^) | | |  | Wheat straw*  (kg ha^-1^) | | | | Irrigation  (mm) | Diesel  (kg ha^-1^) | Seeding rate  (kg ha^-1^) | Pesticides  (kg ha^-1^) |
| --- | --- | --- | --- | --- | --- | --- | --- | --- | --- | --- | --- | --- |
|  | N | P_2_O_5_ | K_2_O |  | Amount | N | P | K |  |  |  |  |
| 2003 | 112.5 | 171.8 | 0.0 |  | 0 | 0 | 0 | 0 | 0 | 15.5 | 37.5 | 2.5 |
| 2004 | 254.3 | 82.5 | 43.4 |  | 0 | 0 | 0 | 0 | 0 | 15.5 | 45.0 | 3.2 |
| 2005 | 168.0 | 82.5 | 43.4 |  | 0 | 0 | 0 | 0 | 150.0 | 15.5 | 45.0 | 3.2 |
| 2006 | 192.0 | 0.0 | 36.2 |  | 0 | 0 | 0 | 0 | 150.0 | 15.5 | 120.0 | 3.0 |
| 2007 | 300.0 | 309.2 | 126.5 |  | 0 | 0 | 0 | 0 | 115.0 | 15.5 | 45.0 | 3.2 |
| 2008 | 207.0 | 0.0 | 0.0 |  | 0 | 0 | 0 | 0 | 0 | 15.5 | 45.0 | 4.2 |
| 2009 | 107.0 | 0.0 | 0.0 |  | 0 | 0 | 0 | 0 | 0 | 15.5 | 28.0 | 3.0 |
| 2010 | 108.0 | 100.5 | 48.0 |  | 7680 | 69.5 | 4.1 | 164.5 | 0 | 15.5 | 30.0 | 2.6 |
| 2011 | 105.0 | 46.5 | 57.8 |  | 0 | 0 | 0 | 0 | 0 | 15.5 | 18.0 | 3.8 |
| 2012 | 285.0 | 31.5 | 36.0 |  | 8377 | 95.3 | 3.6 | 191.3 | 0 | 15.5 | 30.0 | 6.8 |
| 2013 | 187.5 | 150.1 | 45.0 |  | 9211 | 104.7 | 9.1 | 253.4 | 0 | 15.5 | 30.0 | 6.0 |
| 2014 | 374.4 | 118.2 | 48.0 |  | 7290 | 82.9 | 3.1 | 166.5 | 0 | 15.5 | 23.5 | 8.5 |
| 2015 | 150.0 | 29.6 | 60.0 |  | 7836 | 63.9 | 4.9 | 123.8 | 135.0 | 15.5 | 26.3 | 0.8 |
| 2016 | 156.0 | 70.9 | 60.0 |  | 6867 | 56.0 | 4.3 | 108.5 | 0 | 15.5 | 30.0 | 2.7 |
| 2017 | 132.0 | 59.1 | 54.0 |  | 8550 | 93.8 | 5.2 | 136.5 | 0 | 15.5 | 23.3 | 1.7 |
| 2018 | 99.0 | 44.3 | 40.5 |  | 7375 | 80.9 | 4.4 | 117.8 | 0 | 15.5 | 24.0 | 1.8 |
| 2019 | 132.0 | 59.1 | 54.0 |  | 6725 | 73.8 | 4.1 | 107.4 | 0 | 15.5 | 26.0 | 1.8 |
| 2020 | 156.0 | 59.1 | 72.0 |  | 9265 | 107.3 | 9.5 | 226.2 | 150.0 | 15.5 | 26.0 | 3.8 |
| 2021 | 168.0 | 41.4 | 30.0 |  | 8565 | 96.6 | 6.7 | 198.7 | 0 | 15.5 | 26.0 | 3.8 |
| 2022 | 156.0 | 35.5 | 48.0 |  | 10479 | 118.8 | 8.1 | 197.4 | 0 | 15.5 | 26.0 | 3.8 |

Notes: * The straw used to determine the element contents came from the preceding wheat season; ** All from mechanical harvesting.

Table S5 Carbon dioxide equivalent emission coefficients of agricultural input.

| Item | Emission coefficients | References |
| --- | --- | --- |
| Wheat seed | 1.16 kg CO_2_-eq kg^-1^ | Liu et al. (2013) |
| Maize seed | 1.22 kg CO_2_-eq kg^-1^ | Liu et al. (2013) |
| Nitrogen | 8.3 kg CO_2_-eq kg^-1^ | Chen et al. (2014) |
| P_2_O_5_ | 2.332 kg CO_2_-eq kg^-1^ | Chen et al. (2015) |
| K_2_O | 0.66 kg CO_2_-eq kg^-1^ | Liu et al. (2013) |
| Pesticides | 6.58 kg CO_2_-eq kg^-1^ | Liu et al. (2013) |
| Diesel | 3.32 kg CO_2_-eq kg^-1^ | Liu et al. (2013) |
| Electricity | 0.92 kg CO_2_-eq k Wh^-1^ | Liu et al. (2013) |
| σ_1_ | 0.012 kg N_2_O-N of kg^-1^ N applied | Shi et al. (2022) |
| σ_2_ | 0.01 kg N_2_O-N of kg^-1^ N applied | IPCC (2006) |
| σ_3_ | 0.0075 kg N_2_O-N of kg^-1^ N applied | IPCC (2006) |
| *Frac_vol_* | 0.108 kg N volatilized of kg^-1^ N applied | Ge et al. (2011) |
| *Frac_leach_* | 0.3 kg N volatilized kg^-1^ of N applied | IPCC (2006) |

Notes: σ_1_, σ_2_, and σ_3_ mean the emissions factor of direct N_2_O emissions due to N application, the emissions factor for N2O emissions from atmospheric deposition, and the emissions factor for N_2_O emissions from N leaching and runoff of N on soil surfaces, respectively; *Frac_vol_* means the fraction of synthetic fertilizer N that volatilizes as NH_3_ and NO_x_; *Frac_leach_* means the fraction of applied N/mineralized N by the loss of leaching and runoff.

Ref.

Chen, X.P., Cui, Z.L., Fan, M.S., Vitousek, P., Zhao, M., Ma, W.Q., Wang, Z.L., Zhang, W.J., Yan, X.Y., Yang, J.C., Deng, X.P., Gao, Q., Zhang, Q., Guo, S.W., Ren, J., Li, S.Q., Ye, Y.L., Wang, Z.H., Huang, J.L., Tang, Q.Y., Sun, Y.X., Peng, X.L., Zhang, J.W., He, M.R., Zhu, Y.J., Xue, J.Q., Wang, G.L., Wu, L., An, N., Wu, L.Q., Ma, L., Zhang, W.F., Zhang, F.S., 2014. Producing more grain with lower environmental costs. Nature, 514, 486–489.

Chen, S., Fei, L., Wang, X., 2015. Estimation of greenhouse gases emission factors for China’s nitrogen, phosphate, and potash fertilizers. Acta Ecol. Sin. 35 (19), 6371–6383 (in Chinese).

Ge, S., Jiang, Y., Wei, S., Fang, X., 2011. Nitrogen balance under different nitrogen application rates in young apple orchards. Plant Nutr. Fert. Sci. 17 (4), 949–955 (in Chinese).

Liu, X., Xu, W., Li, Z., Chu, Q., Yang, X., Fu, C., University, C.A., University, X.A., University, S.A., 2013. The missteps, improvement and application of carbon footprint methodology in farmland ecosystems with the case study of analyzing the carbon efficiency of China’s intensive farming. Chinese Journal of Agricultural Resources & Regional Planning 34, 1–11 (in Chinese with English abstract).

IPCC, 2006. Guidelines for National Greenhouse Gas Inventories. Institute for Global Environmental Strategies, Japan.

Shi, X., Xiong, J.R., Yang, X.L., Siddique, K. H., Du, T.S., 2022. Carbon footprint analysis of sweet sorghum-based bioethanol production in the potential saline-Alkali land of northwest China. J. Clean. Prod. 349, 131476.

Table S6 The hybrid and growth periods of the wheat season from 2003 to 2022.

| Year | Hybrid | Sowing | Regreening | Jointing | Shooting | Harvest | Growth days |
| --- | --- | --- | --- | --- | --- | --- | --- |
| 2003 | 93-52 | 2002/10/13 | 2003/4/7 | 2003/4/30 | 2003/5/5 | 2003/6/6 | 236 |
| 2004 | Keyu-13 | 2003/10/24 | 2004/2/26 | 2004/4/2 | 2004/4/23 | 2004/6/10 | 230 |
| 2005 | Keyu-13 | 2004/10/10 | 2005/3/15 | 2005/4/7 | 2005/5/2 | 2005/6/15 | 248 |
| 2006 | Wimai8 | 2005/10/29 | 2006/2/19 | 2006/4/5 | 2006/5/2 | 2006/6/11 | 225 |
| 2007 | Taian9118 | 2006/10/6 | 2007/3/1 | 2007/4/4 | 2007/5/2 | 2007/6/7 | 244 |
| 2008 | Kenong199 | 2007/10/25 | 2008/3/2 | 2008/4/9 | 2008/5/1 | 2008/6/10 | 229 |
| 2009 | Kenong199 | 2008/10/16 | 2009/2/18 | 2009/4/1 | 2009/4/25 | 2009/6/9 | 236 |
| 2010 | Wimai8 | 2009/10/17 | 2010/2/25 | 2010/4/16 | 2010/5/8 | 2010/6/16 | 242 |
| 2011 | Laizhou95021 | 2010/10/15 | 2011/3/3 | 2011/4/12 | 2011/5/6 | 2011/6/12 | 240 |
| 2012 | Jimai22 | 2011/10/18 | 2012/3/1 | 2012/4/8 | 2012/4/30 | 2012/6/7 | 233 |
| 2013 | Jimai22 | 2012/10/15 | 2013/2/23 | 2013/4/8 | 2013/5/6 | 2013/6/12 | 240 |
| 2014 | Jimai22 | 2013/10/19 | 2014/3/1 | 2014/4/5 | 2014/4/24 | 2014/6/5 | 229 |
| 2015 | Jimai22 | 2014/10/24 | 2015/3/2 | 2015/4/5 | 2015/5/3 | 2015/6/11 | 230 |
| 2016 | Denghai502 | 2015/10/20 | 2016/3/3 | 2016/4/7 | 2016/4/30 | 2016/6/7 | 231 |
| 2017 | Denghai502 | 2016/10/11 | 2017/3/7 | 2017/4/7 | 2017/4/27 | 2017/6/8 | 240 |
| 2018 | Denghai502 | 2017/10/19 | 2018/3/4 | 2018/4/2 | 2018/4/25 | 2018/6/7 | 231 |
| 2019 | Denghai502 | 2018/10/10 | 2019/3/3 | 2019/4/4 | 2019/4/27 | 2019/6/11 | 244 |
| 2020 | Jimai22 | 2019/10/18 | 2020/3/3 | 2020/4/5 | 2020/4/27 | 2020/6/6 | 232 |
| 2021 | Jimai22 | 2020/10/11 | 2021/2/21 | 2021/3/30 | 2021/4/25 | 2021/6/7 | 239 |
| 2022 | Jimai22 | 2021/11/4 | 2022/2/27 | 2022/4/6 | 2022/4/25 | 2022/6/12 | 220 |

Table S7 The hybrid and growth periods of the maize season from 2003 to 2022.

| Year | Hybrid | Sowing | Jointing | Tasseling | Silking | Harvest | Growth days |
| --- | --- | --- | --- | --- | --- | --- | --- |
| 2003 | Nongda108 | 2003/6/16 | 2003/7/25 | 2003/8/13 | 2003/8/16 | 2003/10/3 | 109 |
| 2004 | Nongda108 | 2004/6/21 | 2004/7/9 | 2004/7/21 | 2004/8/14 | 2004/10/2 | 103 |
| 2005 | Danyu86 | 2005/6/19 | 2005/7/9 | 2005/7/19 | 2005/8/18 | 2005/10/14 | 117 |
| 2006 | Ludan9002 | 2006/6/18 | 2006/7/7 | 2006/7/15 | 2006/8/8 | 2006/9/18 | 92 |
| 2007 | Ludan9002 | 2007/6/14 | 2007/7/8 | 2007/7/22 | 2007/8/12 | 2007/9/24 | 102 |
| 2008 | Zhengdan958 | 2008/6/18 | 2008/7/8 | 2008/7/20 | 2008/8/16 | 2008/9/26 | 100 |
| 2009 | Denghai661 | 2009/6/15 | 2009/6/30 | 2009/7/15 | 2009/8/8 | 2009/9/30 | 107 |
| 2010 | Denghai661 | 2010/6/20 | 2010/7/5 | 2010/7/21 | 2010/8/13 | 2010/10/4 | 106 |
| 2011 | Denghai662 | 2011/6/22 | 2011/7/9 | 2011/7/20 | 2011/8/17 | 2011/10/4 | 104 |
| 2012 | Zhongdan909 | 2012/6/17 | 2012/7/4 | 2012/7/18 | 2012/8/11 | 2012/10/4 | 109 |
| 2013 | Zhengdan958 | 2013/6/15 | 2013/7/3 | 2013/7/21 | 2013/8/9 | 2013/9/28 | 105 |
| 2014 | Zhengdan958 | 2014/6/17 | 2014/7/3 | 2014/7/20 | 2014/8/10 | 2014/10/2 | 107 |
| 2015 | HY1 | 2015/6/18 | 2015/7/11 | 2015/7/20 | 2015/8/16 | 2015/10/2 | 106 |
| 2016 | Yuyu30 | 2016/6/19 | 2016/7/5 | 2016/7/18 | 2016/8/8 | 2016/9/24 | 97 |
| 2017 | Dika517 | 2017/6/17 | 2017/7/5 | 2017/7/19 | 2017/8/10 | 2017/9/29 | 104 |
| 2018 | Jinong1 | 2018/6/15 | 2018/6/29 | 2018/7/18 | 2018/8/4 | 2018/9/26 | 103 |
| 2019 | Dika517 | 2019/6/15 | 2019/7/4 | 2019/7/20 | 2019/8/7 | 2019/9/28 | 105 |
| 2020 | Denghai652 | 2020/6/16 | 2020/7/4 | 2020/7/19 | 2020/8/12 | 2020/9/30 | 106 |
| 2021 | Denghai652 | 2021/6/20 | 2021/7/7 | 2021/7/22 | 2021/8/11 | 2021/10/2 | 104 |
| 2022 | Denghai652 | 2022/6/19 | 2022/7/5 | 2022/7/26 | 2022/8/12 | 2022/10/6 | 109 |

Figure S1 Seasonal variations of cumulative rainfall, mean air temperature, and cumulative sunshine hours in the wheat seasons and in the maize seasons. (wheat season: November to May, maize season: June to September)


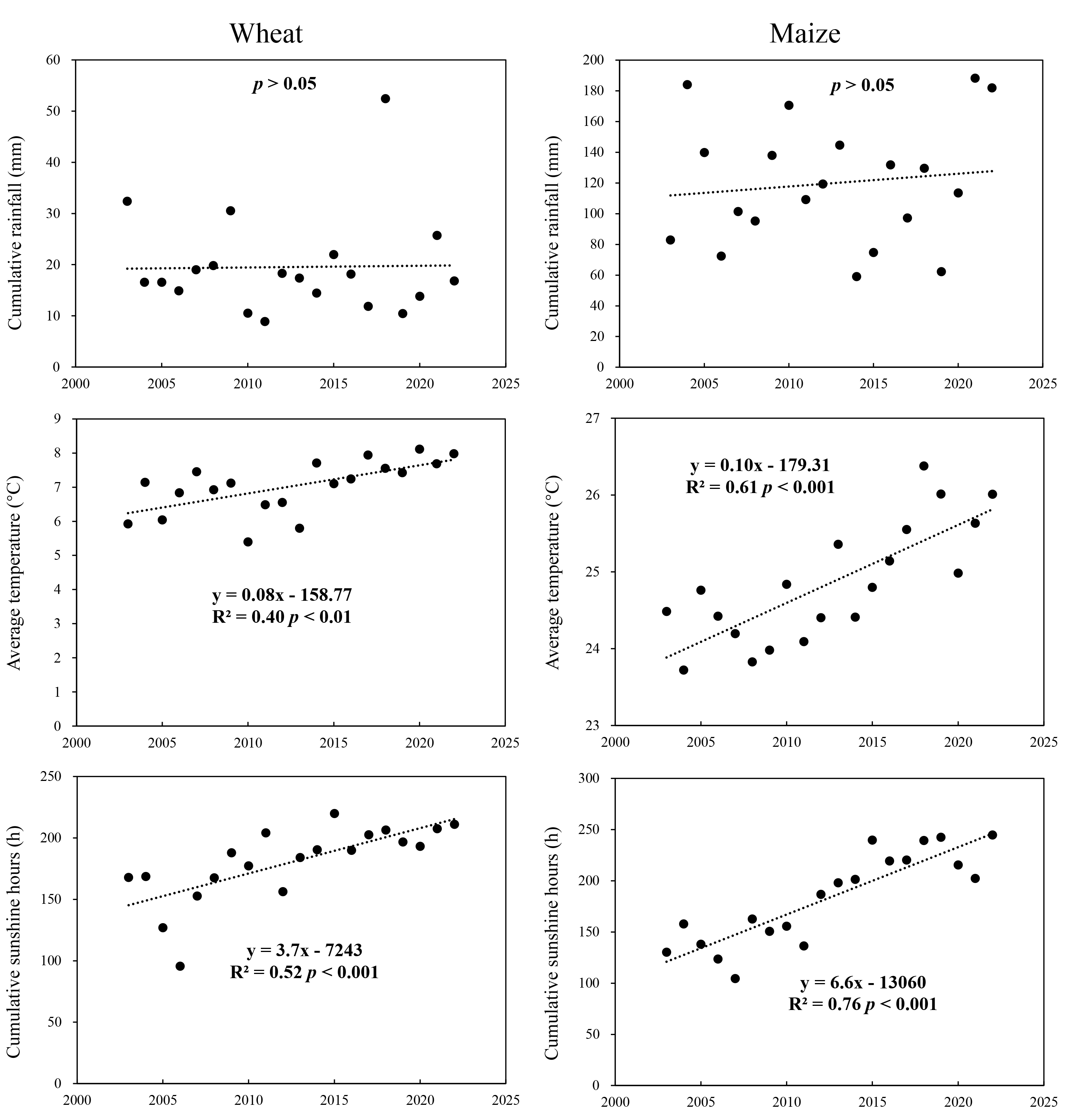


Figure S2 The system boundary for calculating greenhouse gases (GHG) emissions in the winter wheat and summer maize cropping system.


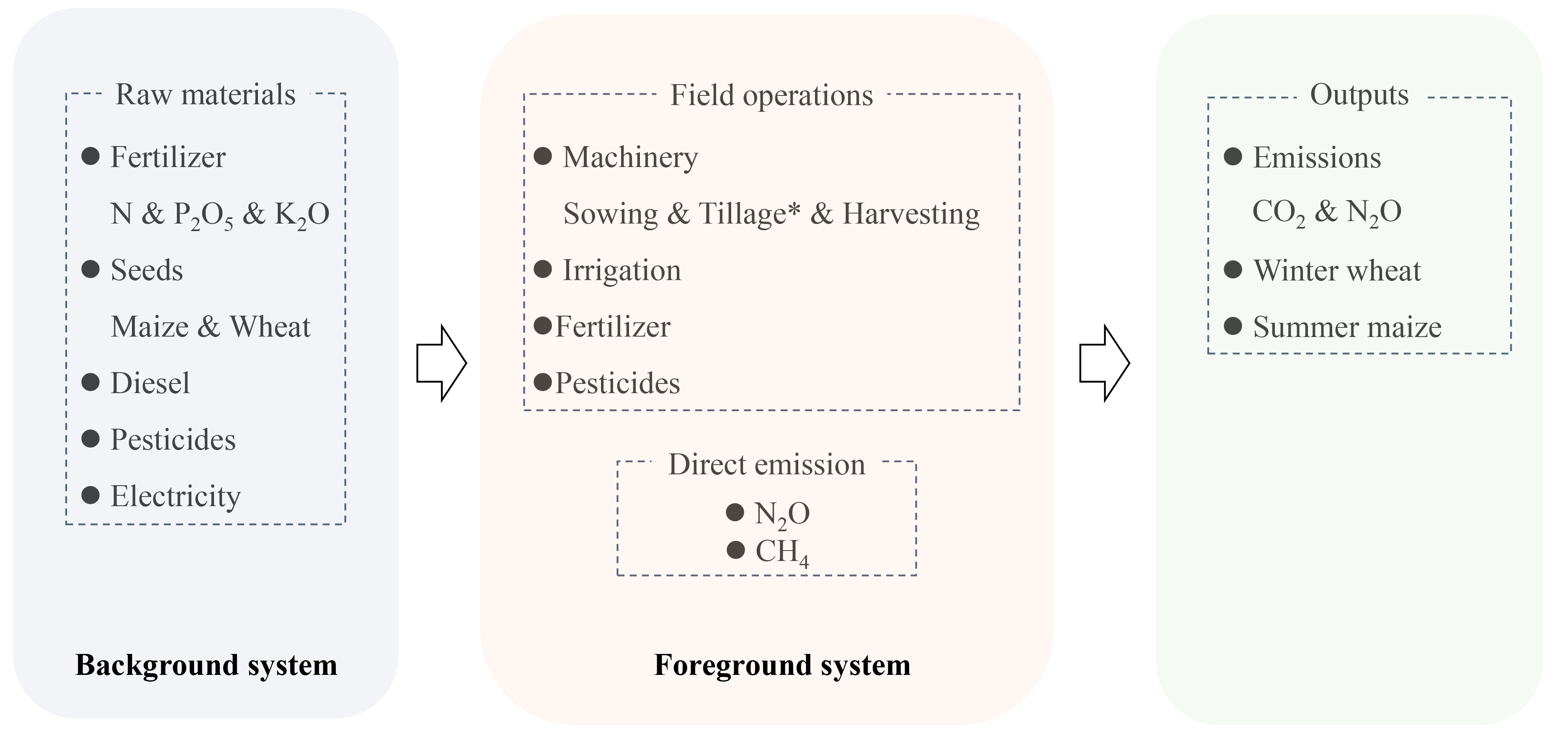


Figure S3 Seasonal variations of soil total N, total P, total K, available N, available P, and rapidly available K in the wheat seasons and in the maize seasons.


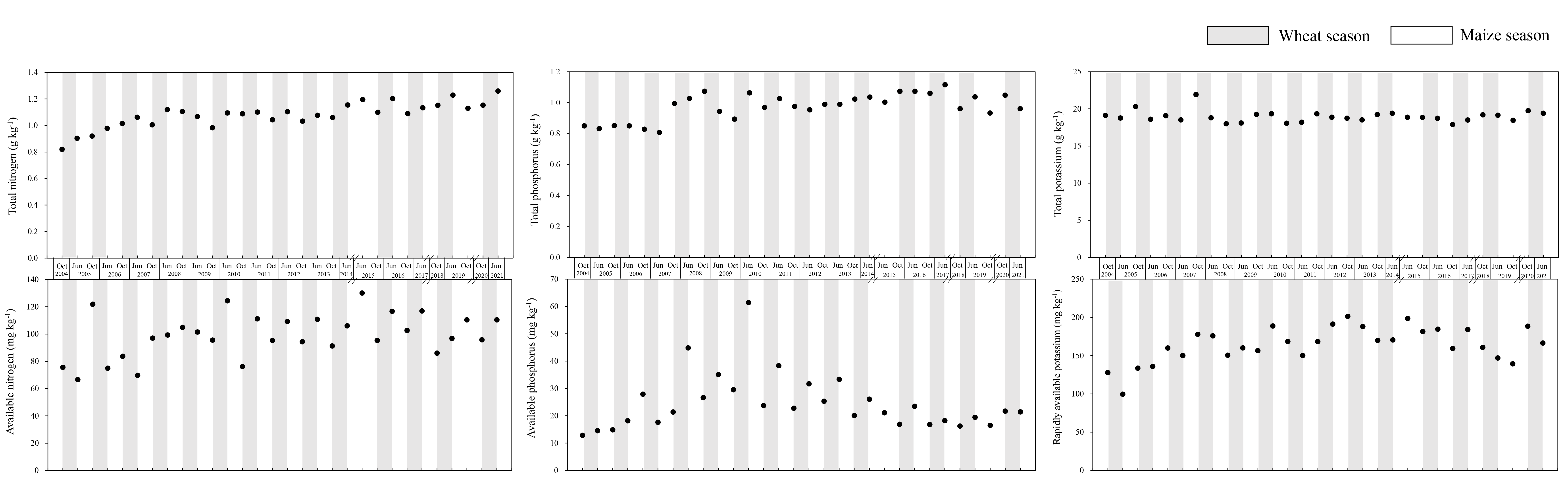

Supplement: Supplementary file 1 [file DataSheet1.docx]
